# Functional MRI examination results in patients with vegetative state: revealing of the Wernicke's area

**DOI:** 10.1186/cc13653

**Published:** 2014-03-17

**Authors:** EA Kondratyeva, SV Diment, SA Kondratyev

**Affiliations:** 1 Russian Polenovs Neurosurgical Institute, St Petersburg, Russia; 2 International Institute of Biological Systems, St Petersburg, Russia

## Introduction

The aim was to evaluate the prognostic importance of the MRI examination using Block Design fMRI in patients in a vegetative state (VS).

## Methods

Twenty-two patients corresponding to the international standards of VS underwent high-resolution MRI (T2 and T1, isomatrix with slice thickness 1.00 mm, T2 FLAIR FS, DWI, SWI) examination and Block Design fMRI (paradigm of passive speech). The patients were aged from 4 to 42 years. Causes of VS: TBI in 19 patients, hypoxia in three patients. By the time of the examination the patients were in VS from 1 to 4 months.

## Results

Wernicke's area activation was observed in nine patients (eight patients with TBI, one with hypoxia). During the subsequent examination, which lasted from 3 to 12 months, seven patients with activated Wernicke's area showed further consciousness expansion up to the minimal consciousness state (further consciousness expansion was seen in two patients). Two other patients with activated Wernicke's area did not show any signs of consciousness. Two patients revealed significant activity in the Broca's area.

## Conclusion

According to the first results of the study one can conclude that behind the outwardly similar clinical symptoms in patients in VS lies a diverse (due to the organization of brain functions) group of patients. fMRI enables one to reveal the first signs of cognitive activity; that is, reveals the linguistic value of speech addressed to the patient which cannot be detected during routine neurological examination.

